# Position Paper on Road Map for RNA Virus Research in India

**DOI:** 10.3389/fmicb.2018.01753

**Published:** 2018-07-31

**Authors:** Guruprasad R. Medigeshi, Katja Fink, Nagendra R. Hegde

**Affiliations:** ^1^Translational Health Science and Technology Institute, Faridabad, India; ^2^Singapore Immunology Network, Agency for Science, Technology and Research, Singapore, Singapore; ^3^National Institute of Animal Biotechnology, Hyderabad, India

**Keywords:** RNA virus, dengue virus, funding models, interdisciplinary, vaccines, clinical trials as topic

## Abstract

The Indian subcontinent with its population density, climatic conditions, means of subsistence, socioeconomic factors as well as travel and tourism presents a fertile ground for thriving of RNA viruses. Despite being pathogens of huge significance, there is very little focus on research into the biology and pathogenesis of RNA viruses in India. Studies on epidemiology and disease burden, risk factors, the immune response to RNA viruses, circulating virus strains and virus evolution, animal models of disease, antivirals and vaccines are strikingly absent. Emerging RNA viruses such as Zika virus, Nipah virus and Crimean-Congo haemorrhagic fever virus are a matter of grave concern to India. Here we summarize the outcome of the India|EMBO symposium on “RNA viruses: immunology, pathogenesis and translational opportunities” organized at Faridabad, National Capital Region, India, on March 28–30, 2018. The meeting focused on RNA viruses (non-HIV), and both national and international experts on RNA viruses covered topics ranging from epidemiology, immune response, virus evolution and vaccine trials concerning RNA viruses. The aim of the symposium was to create a road map for RNA virus research in India. Both concrete and tentative ideas pointing towards short-term and long-term goals were presented with recommendations for follow-up at government level.

## RNA viruses

RNA viruses are amongst the most versatile and complicated pathogens in terms of genetic material and evolution, modes of transmission to various hosts, and persistence in the environment. The diversity of genome architecture and replication strategies by the error prone RNA polymerase drives virus evolution at an unprecedented rate as the viral genomes thrive as quasi-species continuously adapting in response to the host environment (Vignuzzi et al., 2005). Some RNA viruses can exhibit broad host tropism and are known to have the highest propensity to cross species barriers (Geoghegan et al., 2017). This further expands the host range and facilitates emergence of new virus types that cause new diseases, some with the potential to occur as outbreaks and epidemics. Vectors and animal carriers ensure persistence and endemicity of many RNA viruses in most of the tropical and sub-tropical regions of the world. Despite the high disease burden, most of the Low- and Middle-income countries (LMIC), including India, have not invested enough in research and development on RNA viruses. There is very little information on epidemiology and disease burden, risk factors, and the immune response to these viruses in diverse human and animal populations in India. A symposium on “RNA viruses: immunology, pathogenesis and translational opportunities” funded by the European Molecular Biology Organization and Wellcome Trust–Department of Biotechnology India Alliance was organized at Faridabad, National Capital Region, India, on March 28–30, 2018 (http://meetings.embo.org/event/18-rnaviruses). The symposium hosted 6 national and 11 international speakers and chairs, and the participants were from diverse research areas such as virology, bioinformatics, immunology, clinical research, cell and molecular biology, epidemiology, diagnostics and clinical research (please see meeting program in Supplementary Information). Interactive sessions and panel discussions were held to critically analyze the status of RNA virus research in India, the challenges in initiating translational research programs and to explore the way forward. This article presents the outcome of these discussions, which could act as relevant information for funding agencies, institutional leaders and policy makers in India.

## The public health burden of RNA viruses

### Global situation

About 2/3rd of the global population lives in arbovirus-endemic zones and are under risk of exposure to dengue virus (DENV), Zika virus (ZIKV), Chikungunya virus (CHIKV), yellow fever virus (YFV) and/or Japanese encephalitis virus (JEV). DENV alone causes around 60 million apparent cases every year (Stanaway et al., 2016). ZIKV has re-merged in South America and recent reports indicate that changes in the genome have rendered the current circulating strains more virulent (Yuan et al., 2017). A single mutation in CHIKV envelope has enabled replication in a new vector *Aedes albopictus*, thus increasing its geographical range (Tsetsarkin et al., 2007). Enteric RNA viruses such as rotavirus and norovirus continue to be major etiological agents for childhood diarrhea. Most cases of mild and severe respiratory illnesses are by RNA viruses. Respiratory syncytial virus (RSV) is a leading cause of severe pneumonia in children under 2 years of age with close to 33 million infections in 2015 (Shi et al., 2017). The annual epidemics of influenza and the pandemic potential of influenza viruses remain a constant threat. In addition, viruses such as Ebola virus, Crimean-Congo haemorrhagic fever virus (CCHFV) and Middle East respiratory syndrome coronavirus (MERS-CoV) have the potential to lead to outbreaks.

### Indian situation

The burden of viral infections is either not estimated thoroughly and consistently, or is hugely underestimated in India. DENV alone is estimated to cost around $1 billion in terms of annual economic burden (Shepard et al., 2014) and there is no real estimate for infections caused by other RNA viruses such as Chikungunya, influenza, respiratory and enteric RNA viruses. Emerging RNA viruses such as ZIKV, Nipah virus, Chandipura virus, and CCHFV are also a matter of grave concern to India. Tropical weather conditions, poor sanitation and personal hygiene, dense population and lack of prophylactic and therapeutic options primarily contribute to year-round outbreaks and establishment of endemicity for many RNA viruses. Many reports have indicated widespread co-infections but the true impact of co-infections across age groups remains to be determined.

## RNA virus research in india

### Strengths

India now ranks fifth in global research publication output. Vaccine research and development in India is making steady progress, which is evident in the number of product licensures in the last 7 years and the candidate vaccines that are under development (Bhandari et al., 2014; Singh et al., 2015; Dubey et al., 2017; Kulkarni et al., 2017a,b; Sutton et al., 2017; Ramasamy et al., 2018). India now produces more than 60% of the world's vaccines and is a member of the governing council of International Vaccine Institute, with a commitment of US$500,000 every year. The Biotechnology Industry Research Assistance Council (BIRAC) of the Department of Biotechnology has started many initiatives to support research programs on new vaccines and many new programs under BIRAC are nurturing industry-academia partnerships. Similarly, increasing investments by the Indian Council of Medical Research and the Department of Science and Technology have had a significant impact in this direction. The last 10–15 years have seen the establishment of a number of world class educational and research institutions such as the Indian Institute of Science Education and Research (IISERs), Translational Health Science and Technology Institute, National Institute of Biomedical Genomics and National Institute of Animal Biotechnology in addition to several new All India Institute of Medical Sciences and the Indian Institute of Technology. Therefore, the time is apt for creating a roadmap for research on infectious diseases caused by RNA viruses in India.

### Challenges

The challenges for RNA virus research in India are manifold and the bottlenecks, which are further elaborated in this section, could also be relevant for other LMICs.

- ***Manpower:*** Majority of the research activities in India is driven by post-graduate students who join PhD programs or as research fellows supported by extra-mural funds. Postgraduates (Master's degree holders) from over 700 universities in India graduate with minimal research exposure and insufficient skills. Therefore, most research institutes and universities involved in academic research invest large chunk of resources in training doctoral students who go on to pursue their post-doctoral careers outside India due to lack of viable and sustainable career options. A research ecosystem driven by post-doctoral fellows is yet to take hold in India, and there are far fewer quality post-doctoral researchers in Indian Universities and research institutes as compared to western countries.
- ***Training:*** Training in infectious disease research is fractured in India. Basic science researchers do not have exposure to disease biology and disease dynamics in the population. Similarly, clinicians are not trained in infectious disease research and do not acquire skills required to drive an infectious disease research program.
- ***Gaps in knowledge:*** The knowledge of disease burden and the tools required for molecular epidemiology are scarcely available in India. There is not enough ecological research to determine the forces that drive RNA virus infections or their evolution in India. There are no comprehensive programs on diagnostics, surveillance, vaccine development or antivirals, which encompass target identification to proof-of-concept studies.
- ***Interdisciplinarity:*** Discovery and development related to difficult-to-make vaccines, diagnostics and biomarkers requires collaboration among biologists, microbiologists, chemical and material scientists, engineers and clinical researchers. Very few Indian researchers are part of interdisciplinary teams.
- ***Mentorship*:** There is an increasing need to adopt rigor into the academic aspects of RNA virus research in India. Lack of quality mentorship and insufficient critical mass is an impediment to innovation that is relevant and significant at global level.
- ***Vaccine challenges:*** The problems of live-attenuated vaccines for dengue is highlighted by the recent adverse events reported with the only vaccine that is licensed for human use (Tsai et al., 2017). The problem of inaccurate prediction of circulating strains and logistical difficulties in vaccine manufacturing is a challenge for flu vaccines. New vaccines against pathogens such as dengue or influenza viruses need to provide a broad immunity against all serotypes/reassortants. For vaccine candidates like CHIKV, the erratic disease transmission cycle poses a problem for timing the challenge studies to determine efficacy.
- ***Industry-academia partnership:*** The success of vaccine outreach programs need to be backed with quality science before (epidemiology, vaccine development), during (efficacy of the vaccine), and after (effectiveness) deployment of vaccines to ensure the delivery of safe and effective vaccines to the public through these programs. Currently, there is disconnect between the risk taking ability of industry and the academic groups that invest in vaccine development or in generating tools for product development.
- ***Funding:*** Current funding process in India is investigator-centric, providing small grants to large number of researchers for short durations, typically 3 years. There is very minimal interaction between different funding agencies to fund large research programs.

### Enabling translational research

**Figure 1:**
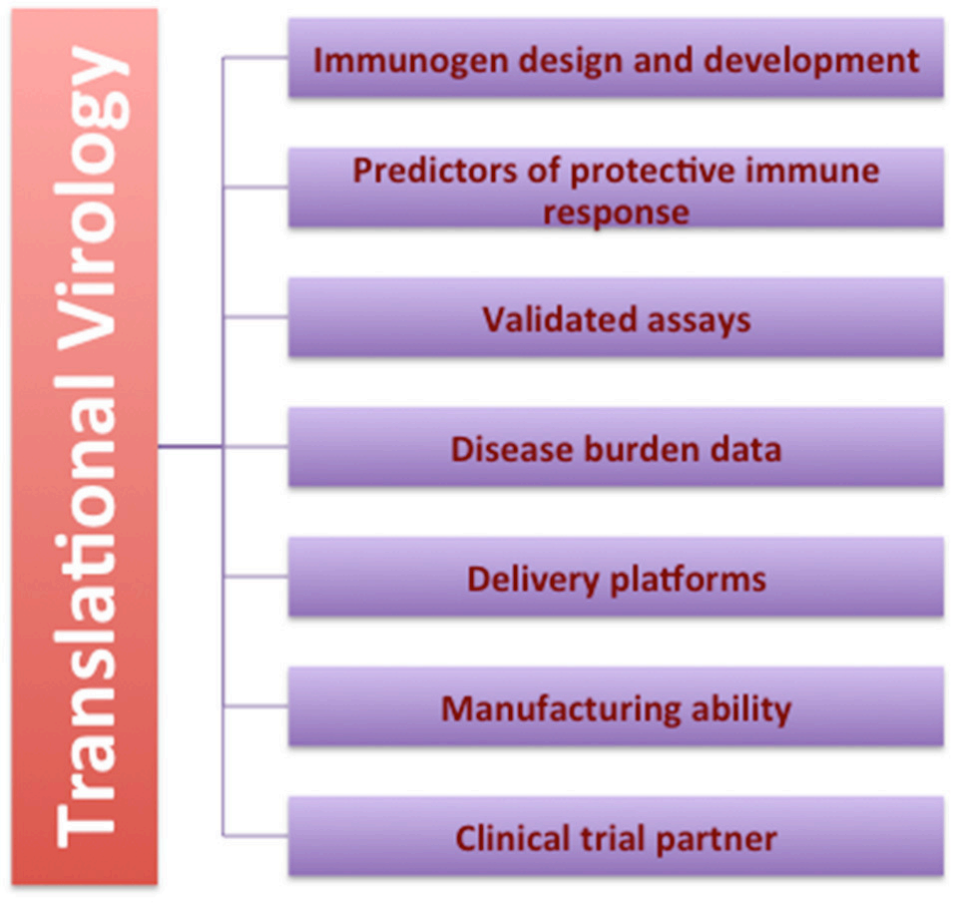
Requirements for a translational virology program. This diagram lists the requirements to initiate, sustain and complete a translational research program in virology. See text for further details.

As compared to the western countries, developing countries like India have been sluggish in building consortia of translational researchers, establishing translational research institutes and funding translational research programs. Although some of the young Indian institutes and researchers are developing research programs with translational outcomes in mind, there is a need to build an enabling ecosystem. For example, as elucidated in Figure 1, translational research program in virology with antiviral or vaccine development as an outcome would require well-designed, sustainable partnerships for:

◦ rational design, optimization and development of immunogens based on the knowledge of epitopes involved in viral entry, molecular interactions, and antigen presentation.
◦ generating definitive knowledge on immune responses that confer protection vs. those that do not.
◦ robust assay platforms that are validated with reference reagents for diagnosis, surveillance, molecular epidemiology and immune responses.
◦ reliable estimation of disease burden from well-designed surveillance studies.
◦ delivery platforms that determine the breadth and longevity of immune responses.
◦ robust manufacturing partners who have low-cost manufacturing capabilities complying with GMP norms and with technical know-how for scale up.
◦ stable clinical trial partners who have a thorough knowledge of regulatory requirements starting from Phase I to post-licensure activities.

As a case in point, development of novel and indigenous vaccine candidate against Dengue (Ramasamy et al., 2018) began in 2009, and, assuming safety and immunogenicity, Phase III trial results are likely to be announced in 2021 or later. Similarly, work began on the structure-guided rational design of candidate influenza vaccine in 2007 and focused and steady progress has led to completion of proof-of-concept studies in animals recently (Bommakanti et al., 2010; Mallajosyula et al., 2014; Sutton et al., 2017). On the other hand, the development of the classical rotavirus vaccine started with an observation in the mid-1980s, and could only be accomplished through a large international consortium working together for over three decades (Bhandari et al., 2014). Thus, enabling translational research, in general, requires a concerted effort, sustained funding and institutional support. Therefore, institutions and funding agencies need to redesign the evaluation and input mechanisms for projects which have translational outcomes as the end point. This is a huge challenge as the investment involved is large and with no short-term outcomes or guaranteed success.

### Way forward

The group of experts at the India|EMBO symposium on RNA viruses debated and recognized the challenges of running sustainable translational program in India and other LMICs. The group also felt that India has unique capabilities and resources to address these challenges and can set an example for other resource-constrained nations. The expert panel and other participants discussed various problems and suggested the following practical solutions that could be implemented as we go forward.

- ***Manpower and training:*** Post-graduate programs (Master's) should integrate research and skill development as part of the curriculum. Post-doctoral programs need to be made attractive in terms of fellowships and independence. Initiatives like the early career fellowships from the Wellcome trust-DBT India Alliance may serve as a good template for other funding agencies. Participation in workshops and symposia of high quality, training in new technologies, grant-writing and communication workshops may ensure quality in doctoral and post-doctoral programs. Specialized courses and training in infectious diseases should be developed for post-graduates in medicine and biology.
- ***Team science:*** To initiate and nurture collaborative endeavors is a challenge in India. There is a need to incentivize interdisciplinary science with translational outcomes as end points. Institutions should create an ecosystem that nurtures individual talent and provide platforms for effective national and global networking opportunities for researchers and clinicians focusing on infectious diseases.
- ***Mentorship:*** Team science needs to be promoted by quality mentors who are globally competitive and are willing to invest their time in building this ecosystem. Offering adjunct faculty positions to acclaimed mentors and facilitating interactions with younger scientists to develop research programs that define the scientific questions with clarity and with a deep understanding for the challenges and probable outcomes will help in building globally competitive research groups. Mentors should be part of scientific advisory groups (SAG) and institutions need to set up an active interaction and mentoring process between the SAG members and young scientists.
- ***Filling gaps in knowledge:*** Deeper insights into virus attachment and entry by structural biology are aiding immunogen design and vaccine development (Mallajosyula et al., 2014; Krarup et al., 2015). Knowledge of the nature of protective immunity, where it is not known, will expedite vaccine development. RNA virus research in India would benefit by focusing on filling the knowledge gaps in areas such as epidemiology, economic burden of the disease, immune response to primary and secondary infections, biology of virus life-cycle with focus on epitopes for vaccine design and affordable point-of-care diagnostics to name a few.
- ***New tools and technologies:*** New strategies and tools are required to address key questions in epidemiology, disease burden, and transmission dynamics at the population level (both human and animal). Capacity building in big data analysis and artificial intelligence can enhance our ability to anticipate and detect threats as well as to plan preventive strategies in time. Implementing new technologies such as Mass cytometry/CyTOF, single cell platforms and organoids may create new opportunities and solutions. Novel vaccines should be capable of inducing rapid host responses in single or two doses, and vaccine platforms should be adaptable to multiple pathogens and should be scalable and easy to manufacture. Vaccine platforms such as the measles vector, modified vaccine Ankara vector, Chimpanzee adenovirus or cytomegalovirus vectors should be explored. Rationally designed subunit vaccines, RNA based vaccines (Scorza and Pardi, 2018) and DNA vaccines (Hasson et al., 2015) are other possible platform approaches.
- ***Partnering with Industry:*** It is imperative that the academic research and development on RNA viruses should be connected to capacity building in vaccine development, scale up in manufacturing and product licensing by the industry. The initiatives taken up by BIRAC should be emulated by other funding agencies and the industry should reciprocate by finding appropriate academic partners to find novel solutions in vaccine manufacturing that could reduce cost, to optimize technology for rapid scale up during outbreaks, and to advance manufacturing processes by automation.
- ***Clinical studies:*** Stable (continuously funded) clinical sites and cohorts are fundamental to building long-term and sustainable research programs. Clinical trial groups need to have an in-depth knowledge of regulatory guidelines and rigorous training to conduct vaccine trials. Organizations such as the Clinical Development Services Agency (CDSA) are playing an important role in providing training to clinicians across the country in this direction. Clinical trial sites should plan biobanking and have a policy of generous sharing of precious samples from cohorts, trials and human challenge studies which will be useful resources for RNA virus research community. The Indo-US vaccine action program and BIRAC may play a critical role in supporting such endeavors. Controlled human infection models (CHIM) are proving to be a game changer for vaccine development (Pollard et al., 2012; Gordon et al., 2017; Jin et al., 2017; Park et al., 2018). India needs to discuss various ethical dilemmas, misconceptions and challenges of CHIM studies and build a platform based on sound regulatory guidelines for including CHIM studies in future vaccine trials. India has a great opportunity among LMICs to take the lead and show the way forward in this regard.
- ***Sustainable funding strategy:*** It is necessary that Indian funding agencies focus on funding long-term programs that address the areas with big knowledge gaps by engaging with researchers. Funding mechanism should be based on calls for proposals in the selected areas of research with mandatory involvement of at least two principal investigators addressing inter-disciplinary questions in RNA virus research. Funds should also be made available to centers for excellence in RNA virus research involving interdisciplinary groups. Emphasis should be laid on the establishment of cohorts with sound study design and long-term follow up plans, along with state-of-the art biobanking facility. Above all, studies on surveillance, diagnostics, molecular epidemiology and disease burden must be supported, so that informed decisions can be made about vaccine development, virus strains, and demographic considerations for vaccine deployment.

## Conclusion

The world today is a single tribe, and viral infections need to be viewed from a global, regional and national perspective. Examples of severe acute respiratory syndrome (SARS), chikungunya and Zika have shown us that viral infections can be local today and global tomorrow. Combating viral infections and outbreaks therefore requires a truly global effort. The community of scientists and concerned professionals must work together with single mindedness to prevent human suffering. We must build capacity and capability in LMICs that are often the most vulnerable to viral infectious diseases, outbreaks and epidemics. India has taken a positive step in partnering with the Coalition for Epidemic preparedness Innovations (CEPI) to anchor a key program for preparing India against emerging viruses. Funding agencies should believe in the philosophy of sustained investment in knowledge and should be actively involved in exchange of ideas with researchers. The leadership of institutions and funding agencies should lend more coherence to the scientific enterprise and provide better direction for utilization of existing resources. We believe these efforts will enhance India's contribution to knowledge generation and innovation in the global RNA virus research arena.

## Author contributions

GM conceived the original idea of the conference. KF and NH were co-organizers of the meeting. All the authors have reviewed the final version of the manuscript.

### Conflict of interest statement

The authors declare that the research was conducted in the absence of any commercial or financial relationships that could be construed as a potential conflict of interest.
