# Supplementary material for: Position Paper on Road Map for RNA Virus Research in India
Source: Front Microbiol. 2018 Jul 31;9:1753. doi: 10.3389/fmicb.2018.01753 (PMC6090158; doi:10.3389/fmicb.2018.01753)
Supplement: Supplementary file 1 [file Image_1.PDF]

India | EMBO Symposium

RNA viruses: Immunology, pathogenesis and translational opportunities

March 28 – 30, 2018, Faridabad, India

Programme

DAY 1 – MARCH 28, 2018

**Session 1: RNA viruses: Epidemiology, disease burden and risk factors**<br>**Chair: V. Ravi, National Institute of Mental Health and Neurosciences, INDIA**	
**9.00-9.15**	Welcome Note<br>**Guruprasad Medigeshi**
**9.15-9.45**	Keynote Address: "Capacity building for RNA virus research in India – ecosystem, challenges and way forward"<br>**Maharaj Kishan Bhan**
**9.45-10.30**	Enteric virus strain diversity: epidemiology and impact on vaccine design and efficacy<br>**Miren Iturriza-Gomara**
**10.30-11.00**	**Coffee break**
**11.00-11.30**	Arboviruses in India: where, when, how much, and what can be done?<br>**Katherine Gibney**
**11.30-11.45**	Complement-mediated neutralization of Chandipura virus is through classical pathway dependent aggregation<br>**Selected abstract: Kunnakkadan, U. et al.,**
**11.45-12.00**	HULC influences HCV replication and release in liver cells<br>**Selected abstract: Geetika Sharma and Saumitra Das**
**12.00-14.00**	**Lunch and poster session**
**Session 2: RNA virus infections: Focus on pathogenesis and evolution**<br>**Chair: Sudhanshu Vrati, Regional Center for Biotechnology, INDIA**	
**14.00-14.45**	MERS-coronavirus: from discovery to intervention<br>**Bart Haagmans**
**14.45-15.15**	Role of complement during the pandemic influenza A(H1N1) 2009 virus infection<br>**Arvind Sahu**
**15.15-15.30**	Uncovering viral surface hotspots in Dengue for targeted antibody discovery<br>**Selected abstract: Anand, G.S. et.al.,**
**15.30-16.00**	**Coffee Break**
**16.00-16.30**	Ebola virus immunology then and now<br>**Cesar Munoz-Fontela**
**16:30-17.00**	Pathogenesis of vascular leak in dengue<br>**Gathsaurie Malavige**
**17:00-17.30**	**Meet the speakers of session 1 and 2**
**17.30-19.00**	**Poster session**
**19.00-21.00**	**Dinner**

DAY 2 – MARCH 29, 2018

**Session 3: Viral and immunological determinants in RNA virus infections**<br>**Chair: Shahid Jameel, Wellcome trust/DBT-India Alliance**	
9.00-09.45	Pandemic Influenza Viruses: Biology and Transmission<br>**Kanta Subbarao**
9.45-10.15	Immune signatures of CD4 T cell subsets in healthy and dengue virus-infected individuals<br>**Daniela Weiskopf**
10.15-10.45	Molecular basis for attenuation of Live attenuated Japanese encephalitis virus vaccine SA14-14-2<br>**Vijaya Satchidanandam**
10.45-11.15	**Coffee break**
11.15-11.45	Human CD8 T cell response in Dengue Virus Infections<br>**Anmol Chandele**
11.45-12.15	Innate immune response to Flaviviruses<br>**Nolwenn Jouvenet**<br>**EMBO Young Investigator Lecture**
12.15-12.45	A systems biology approach to understand immune protection against hepatitis C virus<br>**Fabio Luciani**
12.45-14.00	**Lunch and poster session**
**Session 4: Vaccine development: new strategies for new markets**<br>**Chair: Gagandeep Kang, Translational Health Science and Technology Institute, INDIA**	
14.00-14.45	Crimean-Congo Haemorrhagic Fever (CCHF) Virus - A Growing Threat<br>**Roger Hewson**
14.45-15.15	Designer VLP based tetravalent dengue vaccine candidate<br>**Navin Khanna**
15.15-15.30	Role of immune cell subsets and identification of biomarkers in severe dengue<br>**Selected abstract: Kar, M., et al.,**
15.30-16.00	**Coffee Break**
16:00 - 17:00	Panel Discussion: Road map for RNA virus research in India. Moderator: **Gagandeep Kang.** Panelists: **Bart Haagmans, Kanta Subbarao, Soumen Basak and Shahid Jameel**
17:00-17.30	**Meet the speakers of sessions 3 and 4**
17.30-19.00	**Poster session**
19.00-21.00	**Dinner**

DAY 3 – MARCH 30, 2018

Session 5: Clinical trials and models to test therapies and vaccines<br>Chair: **Saumitra Das, Indian Institute of Science**	
8:30	Departure to NCR Biotech Cluster
9.00-09.45	Understanding Dengue Pathogenesis and Essential Areas for Research<br>**Timothy Endy**
9.45-10.15	Human B cell response to dengue virus infection and lessons for vaccine development<br>**Katja Fink**
10.15-10.45	**Coffee break**
10.45-11.15	Controlled Human Infection Models<br>**Gagandeep Kang**
11.15-11.45	'Women in Science: An Indian Perspective'<br>**Sandhya S. Visweswariah**<br>**EMBO "Women in Science" lecture**
11.45-12.15	**Meet the speakers of session 5**
12.15 -12.45	**Tour of NCR Biotech cluster**
12.45-14.00	**Lunch**
14.00-15.00	**"Crystal gazing" talks by Session chairs - 10 min each**<br>**V. Ravi, Sudhanshu Vrati, Shahid Jameel, Gagandeep Kang, Saumitra Das**
15.00-15.15	Closing Remarks<br>**Nagendra Hegde**
15.15-15.45	**Coffee break and Departure**
